# Genetic Characterizations of *Giardia duodenalis* in Sheep and Goats in Heilongjiang Province, China and Possibility of Zoonotic Transmission

**DOI:** 10.1371/journal.pntd.0001826

**Published:** 2012-09-20

**Authors:** Weizhe Zhang, Xiaoli Zhang, Rongjun Wang, Aiqin Liu, Yujuan Shen, Hong Ling, Jianping Cao, Fengkun Yang, Xiaoyun Zhang, Longxian Zhang

**Affiliations:** 1 Department of Parasitology, Harbin Medical University, Harbin, People's Republic of China; 2 College of Animal Science and Veterinary Medicine, Henan Agricultural University, Zhengzhou, People's Republic of China; 3 National Institute of Parasitic Diseases, Chinese Center for Disease Control and Prevention, Key Laboratory of Parasite and Vector Biology, Ministry of Health, WHO Collaborating Centre for Malaria, Schistosomiasis and Filariasis, Shanghai, People's Republic of China; Universidad Centroamericana, Nicaragua

## Abstract

**Background:**

*Giardia duodenalis* is a widespread intestinal protozoan of both humans and mammals. To date, few epidemiological studies have assessed the potential and importance of zoonotic transmission; and the human giardiasis burden attributable to *G. duodenalis* of animal origin is unclear. No information about occurrence and genotyping data of sheep and goat giardiasis is available in China. The aim of the present study was to determine prevalence and distribution of *G. duodenalis* in sheep and goats in Heilongjiang Province, China, and to characterize *G. duodenalis* isolates and assess the possibility of zoonotic transmission.

**Methodology/Principal Findings:**

A total of 678 fecal specimens were collected from sheep and goats on six farms ranging in age from one month to four years in Heilongjiang Province, China. The average prevalence of *G. duodenalis* infection was 5.0% (34/678) by microscopy after Lugol's iodine staining, with 5.6% (30/539) for the sheep versus 2.9% (4/139) for the goats. Molecular analysis was conducted on 34 *G. duodenalis* isolates based on the triosephosphate isomerase (*tpi*) gene. 29 *tpi* gene sequences were successfully obtained and identified as assemblages A (n = 4), B (n = 2) and E (n = 23). High heterogeneity was observed within assemblage E at the *tpi* locus, with five novel subtypes found out of seven subtypes. Two subtypes of assemblage A were detected, including subtype AI (n = 3) and a novel subtype (designated as subtype AIV) (n = 1). Two assemblage B isolates were identical to each other in the *tpi* gene sequences.

**Conclusions/Significance:**

This is the first report of *G. duodenalis* infections in sheep and goats in China. The present data revealed the unique endemicity on prevalence, distribution and genetic characterization of *G. duodenalis* in sheep and goats in Heilongjiang Province. The findings of assemblages A and B in sheep and goats implied the potential of zoonotic transmission.

## Author Summary

Giardiasis is a kind of zoonotic disease with global distribution. Due to the great number of asymptomatic giardiasis cases, human giardiasis is often underreported. The sources of infection of giardiasis are feces of humans and mammals with the pathogen being transmitted by the fecal-oral route. In this study, we described the occurrence of sheep and goat giardiasis and genetic charaterizations of *G. duodenalis* isolates in Heilongjiang Province, China. The average infection rate was 5.0% (34/678), with 5.6% (30/539) for the sheep versus 2.9% (4/139) for the goats. *G. duodenalis* assemblages and subtypes were genetically diagnosed by sequence analysis. Three assemblages were successfully identified out of 29 *tpi* gene sequences, with the percentages 13.8% (4/29), 6.9% (2/29), 79.3% (23/29) for assemblages A, B and E, respectively. Five novel subtypes were found out of seven subtypes of assemblage E. Two subtypes of assemblage A were detected, with one belonging to a novel subtype and the other belonging to assemblage AI. Two assemblage B isolates were identical to each other at the *tpi* locus. Prevalence, distribution and genetic characteristics of subtypes of *G. duodenalis* in sheep and goats appear to be unique in the areas examined. The sheep and goats infected with assemblages A and B have important public heath significance.

## Introduction

*Giardia duodenalis* (syn. *Giardia lamblia, Giardia intestinalis*) is one of the most common protozoa in humans and animals. Giardiasis of humans and animals has a wide spectrum of clinical signs, including the onset of diarrhea, abdominal pain, bloating, vomiting, nausea and/or weight loss. The parasite can lead to growth and development retardation of children, even in asymptomatic cases [1], and is also of significant clinical and economic importance in livestock and pet animals [2]–[4].

To date, molecular data revealed the presence of seven assemblages (A to G) within *G. duodenalis* based on genetic analysis and host specificity. In a recent study, assemblage H has been described in marine vertebrates [5]. Among them, assemblages A and B have the widest host range. They both have the ability to infect humans and a variety of mammals, including livestock, dogs, cats and wildlife [6], [7]. Assemblages C, D, E, F and G seem to be host specific for nonhuman species. However, assemblages C, D, E and F have been isolated from humans, but at a very low prevalence [7].

Outbreaks of human giardiasis are most frequently waterborne and caused by contamination of drinking water, although other routes have also been described [8], [9]. *G. duodenalis* is one of the most common pathogens in water-associated outbreaks of parasitic protozoan diseases, accounting for 40.6% (132/325) out of total outbreaks [10]. The sources of contamination of water supplies may come from humans, farm animals and wildlife. Due to the lack of data of transmission dynamics between humans and animals, the role of animals in the spread of infections remains unclear. Contact with farm animals has been pointed out as a risk factor of human infection of *G. duodenalis* in case control studies [11], [12]. The previous report of an outbreak of sheep giardiasis with the death of some animals in Central Italy is of concern for sheep farmers [3].

Years of epidemiological data have documented the occurrence of *G. duodenalis* infection in sheep and goats, indicating a wide range in prevalence from 1.5% to 55.6% in sheep and from 12.3% to 42.2% in goats [13]. Studies using molecular analysis have confirmed the presence of assemblages A, B and E of *G. duodenalis* in sheep and goats. Assemblage E was the major genotype in sheep/goats in most countries, with assemblage A being occasionally detected in Australia, Sweden, the USA, Belgium, and Spain and with assemblage B being occasionally detected in Norway and Spain [14]–[21]. In a study in Australia, assemblage A is the most common subtype in sheep [22]. Interestingly, only *G. duodenalis* assemblages A and B have been reported in sheep in Italy, with an absence of assemblage E [3], [23].

In China, there are no reports about the investigations and genotyping data of sheep and goat giardiasis so far. China has one of the largest populations of sheep and goat flocks [24]. To understand the prevalence and distribution of *G. duodenalis* in sheep and goats in China, a survey of *G. duodenalis* was conducted in sheep and goats in Heilongjiang Province, and the *tpi* gene sequences of *G. duodenalis* isolates were analyzed for genetic characterization and assessment of the potential of zoonotic transmission at both genotyping and subtyping levels.

## Materials and Methods

### Ethics Statement

Before beginning work on the study, we contacted the farm owners and obtained their permission to have their animals involved. During specimen collection, all animal work followed guidelines in accordance with the Regulations for the Administration of Affairs Concerning Experimental Animals, and approved by the Animal Ethical Committee of Harbin Medical University.

### Specimen collection

A total of 678 fecal specimens (539 from sheep and 139 from goats) were randomly collected on six farms within Heilongjiang Province, China in a two-year survey from October 2009 to November 2011 (Table 1). The specimens were taken directly from the rectum of each animal with sterile plastic gloves. For each animal, the sampling date, species, ages and the identification number were recorded. Their ages ranged from one month to four years. The specimens were transported to the laboratory in a cool box and then stored at 4°C and processed within two days of collection. Each fecal specimen was directly used to smear three slides for iodine wet mount staining. Wet smears were examined for the presence of *G. duodenalis* cysts by light field microscopy at 400× magnification. All *G. duodenalis*-positive specimens were stored in 2.5% potassium dichromate solution at 4°C before DNA extraction.

**Table 1:** Prevalence and assemblage distribution of *G. duodenalis* on six farms of sheep and goats in Heilongjiang Province.

Farm	Animal species	No. of Positive/No. of Examined (%)					No. of *G. duodenalis* assemblages A, B and E
		Age group (month)					
		<2	3–6	7–11	>12	Total	
Farm1	sheep	4/15 (26.7)	5/54 (9.3)	1/21 (4.8)	1/46 (2.2)	11/136 (8.1)	E(9)
Farm2	sheep	2/11 (18.2)	2/41 (4.9)	1/29 (3.4)	0/23 (0)	5/104 (4.8)	A(1), B(2)
Farm3	sheep	1/6 (16.7)	2/33 (6.1)	2/41 (4.9)	0/10 (0)	5/90(5.6)	E(4)
Farm4	sheep	2/13 (15.4)	4/42 (9.5)	3/58 (5.2)	1/35 (2.9)	10/148 (6.8)	A(3), E(6)
Farm5	sheep	0/8 (0)	0/22 (0)	0/13 (0)	0/18 (0)	0/61 (0)	
Farm6	goat	2/19 (10.5)	1/21 (4.8)	1/48 (2.1)	0/51 (0)	4/139 (2.9)	E(4)
Total		11/72 (15.3)	14/233 (6.0)	8/220 (3.6)	2/183 (1.1)	34/678 (5.0)	A(4), B(2), E(23)

### DNA extraction

*G. duodenalis*-positive fecal specimens were washed four times with distilled water to remove potassium dichromate from the solution. Genomic DNA was directly extracted from approximately 200 mg of the fecal pellet using a QIAamp DNA Stool Mini Kit (QIAgen, Hilden, Germany) according to manufacturer's instruction. DNA was eluted in 200 µL of AE elution buffer and DNA preparation was stored at −20°C prior to use in PCR analysis.

### *G. duodenalis* genotyping and subtyping

*G. duodenalis* isolates were genotyped and subtyped at the *tpi* locus using a nested PCR which amplifies an approximately 530-bp fragment as previously described [25]. All secondary PCR products were purified and directly sequenced. Each DNA preparation was analyzed at least twice by PCR. Genotypes and subtypes of *G. duodenalis* isolates were identified by analyzing and comparing the *tpi* gene sequences obtained in the present study with those published in GenBank.

### DNA sequence analysis

All secondary PCR products were sequenced with secondary PCR primers on an ABI PRISM™ 3730 DNA Analyzer (Applied Biosystems, USA), using a BigDye Terminator v3.1 Cycle Sequencing kit (Applied Biosystems, USA). Accuracy of the sequencing data was confirmed by sequencing in both directions and a new PCR product once more if necessary for some isolates. Nucleotide sequences obtained in the present study were aligned with each other and reference sequences downloaded from GenBank and analyzed using Clustal X 1.83.

## Results

### Prevalence of *G. duodenalis*

678 fecal specimens were examined by microscopy after iodine staining. 34 of which were positive for *G. duodenalis* cysts. The average prevalence of *G. duodenalis* infection was 5.0% (34/678), with 5.6% (30/539) for the sheep versus 2.9% (4/139) for the goats. With the exception of Farm 5 where *G. duodenalis* infection was absent, the parasite was detected on the other five farms (Table 1).

### Assemblage distribution of *G. duodenalis*

PCR products were obtained from 32 of 34 *G. duodenalis* -positive specimens. However, only 29 were successfully sequenced at the *tpi* locus. The sequences obtained were aligned with reference sequences and identified as three *G. duodenalis* assemblages: 13.8% (4/29) for assemblage A, 6.9% (2/29) for assemblage B and 79.3% (23/29) for assemblage E. Assemblage E was not only the most prevalent but also the most widespread in the investigated areas, accounting for assemblage E on four farms, assemblage A on two farms and assemblage B on only one farm (Table 1).

### Genetic diversity of assemblages A, B and E

Sequence analysis of the *tpi* gene of *G. duodenali*s revealed the presence of two subtypes out of four assemblages A isolates. Three assemblage A isolates belonged to subtype AI (JQ928711), which had 100% similarity with the human-derived subtype AI sequences from Malaysia (HQ836660), France (FJ560569), the USA (EF688031 to EF688043), Peru (EF688038, EF688039), Australia (EF688031, EF688034), Israel (EF688040), Egypt (EF688036) and Henan of China (GU564274 to GU564276). It was also identical to subtype AI sequences from animals, including sheep in Australia (GQ444447), cattle (EF654693, AY655704) in the USA, and cats (AB569393) in Japan. The remaining one was a novel subtype of assemblage A (JQ928710), having four, four and five base differences compared to AI, AII and AIII, respectively (Table 2). The genetic variations led to our proposal of the designation of subtype AIV. The same sequences have been found in human-derived assemblage A isolates from Sweden (GQ329677, GQ329678) and Western Sahara (EU041756), and cat-derived assemblage A isolates (AB569394, AB569398) from Japan. In the present study, two sequences of assemblage B were identical to each other at the *tpi* locus (JQ928712) and had 100% homology with those from dairy cattle (JN162353), rabbits (HQ397719) and wastewater (HQ603781) in the investigated areas.

**Table 2:** Variations in the TPI nucleotide sequences among subtypes of *G. duodenalis* assemblage A in sheep and goats in Heilongjiang Province.

Sequence	Nucleotide at position							Accession no. in GenBank
	Subtype	28	41	92	102	210	370	
Ref sequences	AI	T	G	T	T	A	C	GU564274
	AII	T	G	T	C	A	T	GU564277
	AIII	T	G	C	T	A	C	EF654695
This study	AI	T	G	T	T	A	C	JQ928711
	AIV	C	A	T	C	G	C	JQ928710

The intra-genotypic diversity of *G. duodenalis* assemblage E was observed in the present study. Seven representative sequences (JQ928713 to JQ928717, JQ951964, JQ951965) were obtained out of 23 *tpi* gene sequences. They were respectively named as subtypes E-I to E-VII for convenient description, with one to four base variations noted at nine different nucleotide sites (Table 3) using the AY655706 as a reference sequence. The same sequences have been described, with subtype E-I (n = 9) having been found in cattle (AY655706, EF654682, AY228646, JN162347), and subtype E-II (n = 2) in sheep (GQ444461). The remaining five subtypes (E-III to E-VII) were never identical to any reported assemblage E subtypes and all of them had a low frequency, accounting for 13.0% (3/23) for E-IV and EVII, and 8.7% (2/23) for EIII, E-V and E-VI.

**Table 3:** Variations in the TPI nucleotide sequences among subtypes of *G. duodenalis* of assemblage E in sheep and goats in Heilongjiang Province.

Subtype (No.)	Nucleotide at position									Accession no. in GenBank
	45	78	82	108	262	303	430	441	469	
Ref sequence	T	T	A	C	T	T	A	G	G	AY655706
E-I(9)	T	T	A	C	T	T	A	G	G	JQ928713
E-II(2)	T	T	G	C	T	T	A	A	G	JQ928714
E-III(2)	C	G	A	T	T	T	G	G	G	JQ928715
E-IV(3)	C	T	G	C	T	T	A	G	G	JQ928716
E-V(2)	T	T	G	C	C	T	A	A	G	JQ928717
E-VI(2)	T	T	A	C	T	T	A	G	A	JQ951964
E-VII(3)	T	T	A	C	T	C	A	G	G	JQ951965

In the present study, even on some individual farms, we also observed the widespread occurrence of intra-genetic diversity of assemblage E at the *tpi* locus. At least two subtypes have been found on four farms for 23 isolates of assemblage E. For assemblage A, two subtypes representing four isolates have been found on Farm 2 and Farm 4, respectively (Table 4).

**Table 4:** *G. duodenalis* genotypes and subtypes identified in sheep and goats on farms in Heilongjiang Province.

Farm	No. of Positive	Subtype (No.)		
		Assemblage A	Assemblage B	Assemblage E
Farm1	9			E-I(5), E-II(2), E-III(2)
Farm2	3	AIV(1)	B*(2)	
Farm3	4			E-I(2), E-VI(2)
Farm4	9	AI(3)		E-IV(3), E-VII(3)
Farm5	0			
Farm6	4			E-I(2), E-V(2)
Total	29	AI(3), AIV(1)	B*(2)	E-I(9), E-II(2), E-III(2), E-IV(3), E-V(2), E-VI(2),E-VII(3)

* There is no clear subtyping in assemblage B currently.

## Discussion

The present study is the first to report the occurrence and genetic characterization of *G. duodenalis* in sheep and goats in China. The average infection rate for sheep would be 5.6% versus 2.9% for the goats, which was lower than those reported worldwide except in Italy (1.5%) [13]. Infection rates of *G. duodenalis* were noticed to be inversely associated with the age of animals, with the highest infection rate (17.0%) in lambs and (10.5%) in goat kids. There were an apparent declining infection rates with the increasing age of the animals (Table 1). Young animals were more susceptible to opportunistic parasites than adults. Thus, in the limited epidemiological studies of giardiasis of sheep and goats, the majority focused on the occurrence of *G. duodenalis* in lambs and goat kids. In a longitudinal study of lambs in Norway, overall prevalence of *G. duodenalis* was 23.0% at the first sampling and 31.0% at the second sampling [19]. In Belgium, the prevalence was 25.5% in lambs and 35.8% in goat kids [14]. A 42.0% infection rate of *G. duodenalis* was reported in (one to three)-month-old lambs in Spain [20]. In fact, infection rates are complicated and are often related to many factors, including detection methods, health status and age of the animals, size and structure of specimens, management system, geographical and seasonal differences [14]. Due to the lack of related epidemiological data on giardiasis of animals in the investigated areas, we could not give the reasons for the low prevalence in addition to the low sensitivity of morphological methods and the relative small number of specimens examined from less than two-month-old lambs and goat kids compared to other age groups in the present study. Four out of five sheep farms and the one goat farm had *G. duodenalis* infected animals, and infection rates ranged from 2.9% to 8.1% (Table 1). Farm 5 was not contaminated by *G. duodenalis*. This might be related to strict sanitation management with the farm being built newly.

Currently, PCR-based molecular analysis techniques (DNA sequencing of PCR products and PCR-RFLP) have been developed and used to identify *G. duodenalis*-positive isolates [26]. Among the numerous target genes (such as the SSU rRNA, *gdh*, *tpi*, *ef1α*, *bg*, and variant surface protein [*vsp*] genes), *tpi* gene was frequently used to differentiate *G. duodenalis* at genotype and subtype levels because of the highest genetic heterogeneity at the locus [27]. In the present study, 29 partial *tpi* gene sequences were obtained, with 13.8% (4/29),6.9%(2/29) and 79.3% (23/29) for assemblages A, B and E, respectively. Assemblage E was detected on four farms, whereas assemblages A and B were detected on two farms and one farm, respectively (Table 1). Assemblage E was more prevalent and widespread than assemblages A and B in sheep and goats in the present study. Similar results were also commonly seen in sheep/goats, respectively in Belgium, Australia, Sweden, the USA, Norway, Spain and Mexico, where molecular epidemiological data have showed that assemblage E constituted the majority of *G. duodenalis*-positive specimens (percentages from 60.7% to 100%) [14]–[21], [28], [29]. Assemblage E seems to have apparent host preference. Molecular epidemiological data showed that assemblage E is the most common in cattle and pig in addition to sheep and goats [13]. The present results implied that sheep and goats in the investigated areas have been infected with assemblage E and posed a threat to other susceptible animals, including uninfected sheep and goats, cattle and pigs. Assemblage E might be less risky to humans based on the fact that it has only been isolated from three Egyptians [30]. It has been reported that *G. duodenalis* infections have an effect on growth and performance of animals, and most of relationship studies are only at the species level of *G. duodenalis* [31]. A recent study demonstrated that lambs infected with *G. duodenalis* could lead to decreased hot carcase weights and dressing percentage of sheep [32]. Up to date, no information is obtained on clinical symptoms of animal giardiasis resulting from assemblage E.

Sequence analysis of the *tpi* gene of *G. duodenalis* revealed the presence of seven subtypes out of 23 assemblage E isolates. The most common subtype E-I (39.1%; 9/23) showed 100% similarity with the cattle-derived assemblage E sequences in our investigated areas (JN162347) and the USA (AY655706, EF654682, AY228646). Subtype E-II (8.7%; 2/23) had the same nucleotide sequence as the isolate of *G. duodenalis* from an Australian sheep (GQ444447). Subtypes E-I and E-II appeared to have no differences in geographical distribution. The remaining five novel subtypes (E-III to E-VII) have never been reported before and might represent endemic genetic characterizations of assemblage E in the investigated areas. In addition to high polymorphism observed within assemblage E in sheep and goats at the *tpi* locus in the present and previous studies [29], intra-genotype variations of assemblage E were also described in the animals at the *bg* locus [14], [20], [29]. A high level of genetic diversity within assemblage E has also been reported in cattle based on the *tpi* gene [27], [33].

It is generally considered that assemblages A and B are infrequently detected in sheep and goats. However, there are exceptions appearing in a few studies of sheep and goat giardiasis, with assemblage A being found to be a common genotype besides assemblage E [14], [17], [18]. Even in an Australian study, assemblage A was found to be more prevalent than assemblage E in sheep [22]. More surprisingly, assemblage A isolates have been identified from Italian sheep with the absence of assemblages B and E [23]. For assemblage B, so far, there have only been four reports involved in sheep and goats, with three in sheep [3], [19], [21] and one in goats [34]. The low occurrence of assemblages A and B in sheep and goats as well as in cattle may be related to the predominance of assemblage E in these animals, for they have to compete with the more common assemblage E.

In the present study, four assemblage A isolates (13.8%; 4/29), representing two subtypes, were detected from the *G. duodenalis*-positive specimens. Three of them were identified as subtype AI, showing 100% homology with each other as well as the subtype AI *tpi* gene sequences from humans (Table 5) and some animals [22], [27], [35], [36]. The remaining one isolate was a novel subtype of assemblage A, which was designated as subtype AIV based on the base variations compared to subtypes AI, AII and AIII, respectively (Table 2). The same sequences have been obtained from humans in Sweden and Western Sahara (Table 5), and cats in Japan [36]. Currently, three subtypes AI, AII and AIII constitute the overwhelming majority of assemblage A isolates, and all of them appear to have different host preferences. Subtype AI is mostly found in animals and occasionally in humans; subtype AII commonly infects humans and was sometimes seen in animals; subtype AIII circulated in wildlife, but it has been seen in a few humans based on the *bg* gene [5]. Two *tpi* gene sequences of assemblage B isolates in the present study had 100% homology with each other and was identical to those obtained from dairy cattle, rabbits and wastewater in the investigated areas [33], [37], [38]. The same sequence has never been reported before in human and animal cases in other countries or areas. This may be of characteristic geographical distributions.

**Table 5:** Homology analyses of sheep/goat-derived isolates of assemblages A and B with human-derived isolates.

Country	Ref	Host	No. of isolates characterized	Loci amplified	Assemblage (subtype)	Accession No.	Accession No. of human-derived isolates^b^ (country)
Australia	[22]	Sheep	26	TPI	A (AI)	GQ444447	EF688042-43(USA), EF688041(Puerto Rico), EF688040(Israel), EF688036(Egypt), EF688038-39(Peru), EF688031,34(Australia), GU564274-76(China), HQ836660(Malaysia)
		Sheep	1	TPI	A	GQ444448	No
		Sheep	1	TPI	A	GQ444449	No
		Sheep	1	TPI	A	GQ444450	No
		Sheep	1	TPI	A	GQ444451	No
unspecified	unpublished	sheep	unspecified	GDH	A (AI)	JF958092	AB569385(Japan), EU594662(Cuba), EF685702(USA), GQ502960(Uganda), GQ168944(Australia), GQ329674(Sweden), HM748043(Thailand), JF917089(Iran), JF918516 and JF918453(India)
Belgium	[14]	Sheep	2	BG	A (AII)	EU642896	GU396696 and FJ009208(Poland), HM165227(Sweden)
	[14]	Goat	6	BG	A (AII)	EU642897	No
USA	[16]	Sheep	1	SSU rDNA	A	AY655700^a^	AY826204-05(Holland)
Italy	[23]	Sheep	5	GDH	A (AI)	M84604^a^	No
				BG	A (AI)	M36728^a^	No
Spain	[20]	Sheep	1	BG	A (AI)	EU726988	No
Norway	[19]	Sheep	1	BG	B	GQ337974	No
China	This study	Sheep	1	TPI	A (AIV)	JQ928710	EU041756(Western Sahara), GQ329677-78(Sweden)
			3	TPI	A (AI)	JQ928711	EF688031, 34(Australia), EF688036(Egypt), EF688038, 39 (Peru), EF688040(Israel), EF688041(Puerto Rico), EF688042-43(USA), J560569(France), GU564274-76(China), HQ836660(Malaysia)
			2	TPI	B	JQ928712	No

Note:

a Accession Nos indicating that sheep-derived isolates have 100% homology with the sequences of instead of their own.

b All the human-derived isolates having 100% homology with sheep/goat-derived isolates of assemblages A and B.

Although no human giardiasis cases were reported in the investigated areas, the molecular epidemiological data worldwide can help us to assess the possibility of zoonotic giardiasis caused by sheep and goats. To date, at least 52 sheep-/goat-derived isolates of assemblages A and B have been obtained based on *tpi*, *gdh*, *bg* and SSU rRNA genes. Among 14 representative sequences of assemblage A obtained from 49 isolates, six sequences show 100% homology with those derived from humans. Even in Australia and China, the same sequences of assemblage AI have been seen in both humans and sheep (Table 5). Thus, there might be the large possibility of cross-species transmission of assemblage AI between sheep and humans due to the similar genetic backgrounds of assemblage AI. However, what portion of human subtype AI infections attributable to zoonotic transmission is still unclear. The role that sheep and goats may play in the epidemiology of human giardiasis remains controversial. Some studies do not support sheep and goats as an important reservoir mainly based on the fact that *G. duodenalis* assemblage E is the predominant genotype in these animals in most countries and areas [15], [19], [29]. Up to date, few epidemiological studies have assessed the importance of zoonotic transmission of *G. duodenalis*. An assessment of zoonotic transmission had better come from the dynamics data of giardiasis between humans and animals in the same household or localized focus of endemicity.

In conclusion, the findings above provide the first report on sheep and goat giardiasis in China. Percentages and genetic characteristics of *G. duodenalis* assemblages in Heilongjiang Province seem to be different from other countries or areas and may represent the endemicity of *G. duodenalis*. The fact that the sequences of subtypes AI and AIV in the present study have also been described in human-derived *G. duodenalis* isolates implies the sheep infected with assemblage A posed a serious threat to local inhabitants and are of public health importance. The unique finding of the *tpi* gene sequence of assemblage B in different hosts (cattle, rabbits and sheep) and environmental specimen (wastewater) in the investigated areas may reflect characteristic geographical distribution. The transmission dynamic of assemblages A and B, and the burden of human giardiasis caused by animals need to be assessed by systematic molecular epidemiological investigations of humans and animals in the future.
